# Author Correction: Kriging-based surrogate data-enriching artificial neural network prediction of strength and permeability of permeable cement-stabilized base

**DOI:** 10.1038/s41467-026-73442-0

**Published:** 2026-05-22

**Authors:** Xiaoming Wang, Yuanjie Xiao, Wenqi Li, Meng Wang, Yanbin Zhou, Yuliang Chen, Zhiyong Li

**Affiliations:** 1 School of Civil Engineering, Central South University, Changsha, China; 2 Ministry of Education (MOE) Key Laboratory of Engineering Structures of Heavy Haul Railway (Central South University), Changsha, China; 3 The Second Xiangya Hospital of Central South University, Changsha, China; 4 Hunan Communications Research Institute CO., LTD., Changsha, China

**Keywords:** Civil engineering, Hydrology, Structural materials, Computational methods

Correction to: *Nature Communications* 10.1038/s41467-024-48766-4, published online 7 June 2024

In the version of the article initially published, the source data file provided was an incorrect and less complete version than is now available alongside the article. Below, the authors provide further description of how the Fig. 6d results (building a statistical distribution model for known data) were obtained.

The more data used to calculate the mean and variance, the more accurate the results would be. When calculating the mean and variance of the unconfined compressive strength for the (10, 200) combination in Table 3 (laboratory test results for the uniaxial compressive strength, porosity, and coefficient of permeability of PCBMs), we included an additional nine datasets of mechanical strength obtained from external source with the same design parameters to enhance the accuracy of the results. The mechanical strength values (in units of MPa) obtained from our own laboratory tests are 5.62, 7.58 and 8.58, whereas the additional nine values collected from external source are 6.45, 6.35, 6.97, 6.43, 6.82, 6.80, 6.72, 6.75 and 6.88. It is worth noting that these nine additional values of mechanical strength were obtained from external experiments conducted independently by our industrial partner with identical design parameters. Thus, for the (10, 200) combination, the mean of the 12 (3+9) values of mechanical strength was calculated as 6.83 MPa, and the variance was calculated as 0.69 MPa. Notably, owing to the relatively greater number of unconfined compressive strength data points for the (10, 200) combination, the population variance formula $\sqrt{\frac{1}{n}\sum {({x}_{i}-\bar{x})}^{2}}$ was used, where $n$ represents the number of data points, ${x}_{i}$ represents the values of individual data points, and $\bar{x}$ represents the mean of the data. For other groups with only three data points, the sample variance formula $\sqrt{\frac{1}{n-1}\sum {({x}_{i}-\bar{x})}^{2}}$ was used.

