# Supplementary material for: Kriging-based surrogate data-enriching artificial neural network prediction of strength and permeability of permeable cement-stabilized base
Source: Nat Commun. 2024 Jun 7;15:4891. doi: 10.1038/s41467-024-48766-4 (PMC11161528; doi:10.1038/s41467-024-48766-4)
Supplement: Supplementary file 1 — Supplementary Information [file 41467_2024_48766_MOESM1_ESM.pdf]

Supplementary Information

1. Laboratory test procedures

According to the laboratory experiment requirements, cement and water were thoroughly blended (Supplementary Fig. 1a, b) for the preparation of PCBM samples. Supplementary Fig. 1b shows that for different cement contents, the aggregate surface is uniformly coated with cement paste, and the thickness of the coating increases with increasing cement content. However, when the cement content reaches 20%, slight paste spalling occurs on the aggregate surface due to excessive cement paste. As the cement content increases, the cement film formed on the aggregate surface becomes thicker, leading to a larger contact area of cement bridge between the aggregates and thus stronger specimens after shaping. However, the effective permeable pores may be significantly reduced. The fully mixed mixture is directed into a detachable mold, and static compaction force is applied. The designed static compaction forces are 100 kN, 150 kN, and 200 kN, and after the design compaction force is reached, the molded object is left for 5 minutes before being removed from the press machine. The samples prepared by the static pressure method were allowed to stand at room temperature for 24 hours before demolding. The demolded samples were then placed in a standard curing room for 28 days and removed for further testing (Supplementary Fig. 1c). Finally, an unconfined compression strength test was conducted using a universal testing machine to obtain the unconfined compression strength of PCBM under different compaction forces and cement content conditions.

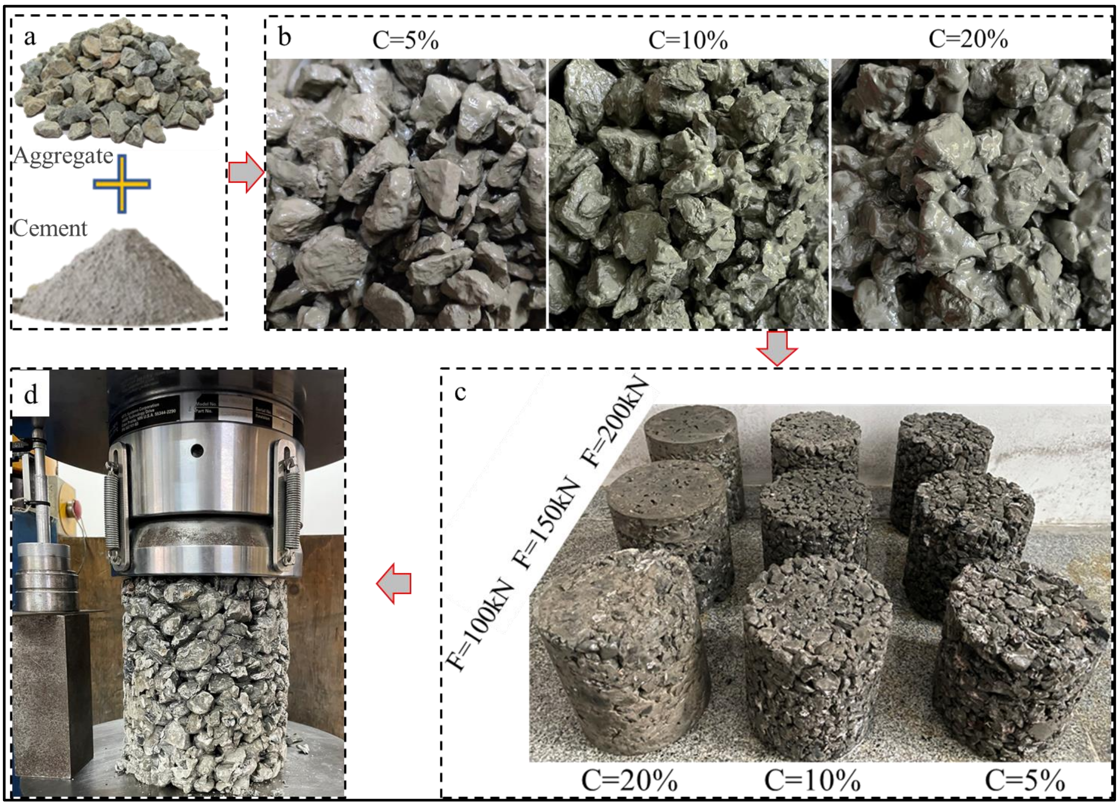

Supplementary Fig. 1 Preparation and unconfined compression tests of permeable cement-stabilized base material (PCBM) specimens. a. Mixing aggregates and cement. **b**. Adhesion state of cement slurry on the surface of aggregates under different cement contents. **c**. PCBM samples prepared under different compaction forces and cement contents combinations. **d**. Conducting unconfined compressive strength tests on PCBM

To measure the porosity of the PCBM, the samples that were cured placed in an indoor environment at an average temperature of 30 ℃ for 72 hours to allow the free water to evaporate completely. Next, the dry weight and height of all the samples were measured. Finally, the samples were placed in water and their buoyant weights were measured (as shown in Supplementary Fig. 2). Each sample was tested three times, and the data were recorded. The average of the three tests' data was taken, with three parallel samples for each condition combination. The porosity of the samples was subsequently calculated using formula (1).

$$P = [1 - \frac{4(w_1 - w_2)}{\rho_w \pi D^2 L} \tag{1}$$

where P represents the porosity of the sample, in %. w_1 represents the dry weight of the sample, in kg. w_2 represents the weight of the sample submerged in water, in kg. ρ_w represents the density

of water, in $kg \cdot m^{-3}$. D represents the diameter of the sample, in m, and L represents the height of the sample, in m.

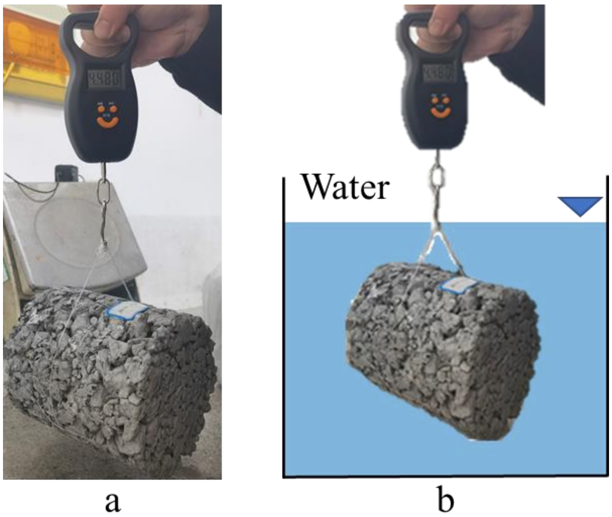

Supplementary Fig. 2 Measurement method for sample porosity (weighing method). a. Dry weight of the sample was measured. **b**. Buoyant weight of the sample was measured.

To determine the permeability performance of the PCBM samples, the constant-head method was employed to measure their permeability coefficient, as illustrated in the model design presented in Supplementary Fig. 3a. The diameters of the overflow ports at the top and bottom of the model are designed to be 30 mm, ensuring smooth water flow during experimentation without any blocking phenomena. According to the design, the constant head permeameter shown in Supplementary Fig. 3b was self-made. The instrument is divided into two parts, upper and lower, connected by a flange at the mid-section with a waterproof rubber pad to prevent water overflow. The internal diameter of the instrument is 145 mm, which is slightly larger than the diameter of the sample (140 mm). A stage was constructed 70 mm below the flange connection position of the instrument to allow for smooth placement of the specimen within the instrument. The height between the top of the instrument and the platform (h) was 380 mm. To ensure that the water flows through all the interconnected pores of the specimen and to eliminate measurement errors caused by boundary effects, the specimen is first tightly wrapped with multiple layers of cling film to prevent water from overflowing from the specimen's boundary. Then, multiple layers of aluminum foil are wrapped around the outer layer of the cling film to achieve maximum sealing around the specimen. The specimen was then placed in the permeameter (Supplementary Fig. 3c) and the top boundary of the

specimen was sealed with sealant to prevent water from flowing out of the gaps between the aluminum foil and the instrument wall. The sealant was fully cured for approximately 30 minutes. Therefore, the permeation test was conducted after the specimen was allowed to stand at room temperature for 30 minutes (Supplementary Fig. 3d). Three tests were conducted for each specimen, and the data were recorded and averaged. Three parallel specimens were tested for each working condition combination. The permeability coefficient of the specimens was calculated using Equation (2).

$$\begin{cases} K = \dfrac{4QL}{\pi D^2 h} \\ Q = \dfrac{\Delta V}{\Delta t} \end{cases} \tag{2}$$

where K represents the permeability coefficient of the specimen, with units measured in $\mathrm{mm \cdot s^{-1}}$. Q represents the flow rate per unit time, in $\mathrm{mm^3 \cdot s^{-1}}$. L represents the height of the specimen, in mm. D represents the diameter of the specimen, in mm. h represents the head height of the water, in mm, where $h = 380mm$. ΔV represents the volume of water flowing out of the bottom overflow spout in Δt time, where the unit of ΔV is mm^3 and the unit of Δt is s.

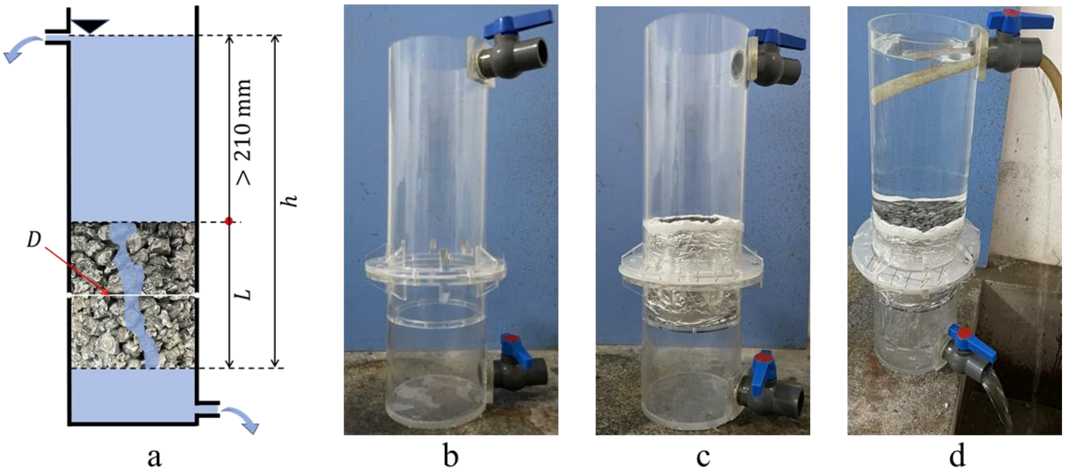

Supplementary Fig. 3 Measuring device and measurement process for the permeability coefficient of the permeable cement-stabilized base material (PCBM) specimen (constant water head method). a. Model diagram of the PCBM penetration test. **b**. Actual instrument made for measuring the PCBM permeability coefficient. **c**. PCBM sample was loaded into the instrument and the gap between the sample and the wall was sealed. **d**. Water was slowly introduced into the instrument, and the flow rate at the overflow outlet at the bottom of the instrument was measured once the water flow stabilized.

2 Laboratory test results and discussion

2.1 Unconfined compression test results

After conducting an unconfined compression test, the stress–strain curve range of the PCBM was obtained and is depicted in Supplementary Fig. 4. As shown in the figure, under the same cement content conditions, the higher the static compaction force applied during specimen preparation is the greater the unconfined compressive strength exhibited by the specimens. This implies that under the conditions of the same thickness of cement paste on the surface of the aggregates, a greater compaction force can lead to the formation of more cement bridges between aggregates, and resulting in a larger area of contact between the cement bridges. This is the primary reason for the increase in unconfined compressive strength observed in the PCBM tests. The unconfined compressive strength of the specimen increases with increasing amount of cement added under the same compaction force. This observation suggested that a thicker mortar coating, achieved by adding more cement, can enhance the bonding between aggregates and form additional cement bridges. Consequently, the overall strength of the specimen increases. When the cement content is 20%, the uniaxial compressive strength of the specimen is greater than 5 MPa under any compaction force. Obviously, there is an excess of unconfined compressive strength for the PCBM. An excessive cement content may significantly reduce the porosity of concrete, thereby adversely affecting its permeability. Consequently, both increased specimen compaction force and increased cement content have an impact on the geometric parameters of the cement bridge. These geometric parameters play a significant role in determining the strength of the sample. Therefore, it is crucial to pay sufficient attention to the optimization design of PCBMs, considering that geometric parameters play a controlling role in the strength of cement bridges.

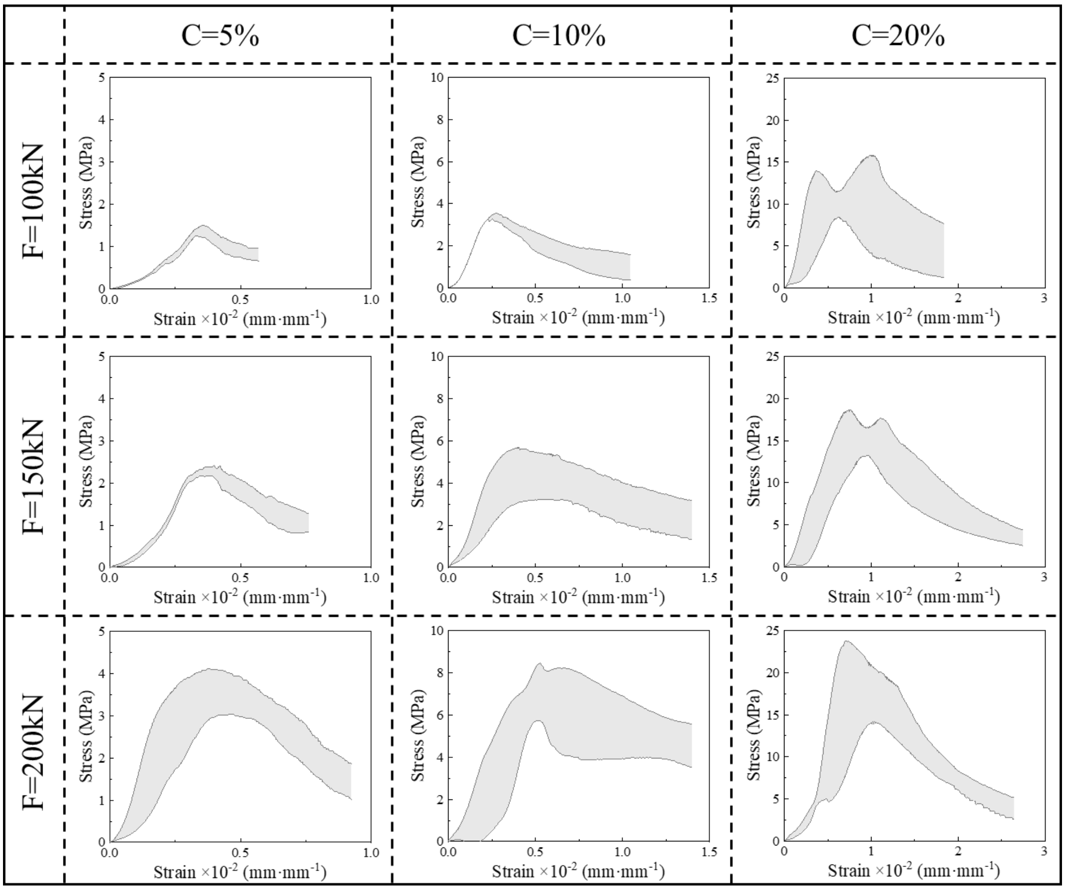

Supplementary Fig. 4 Stress–strain results of permeable cement-stabilized base material (PCBM) under different specimen preparation conditions

Supplementary Fig. 5 shows the typical failure modes of the specimens under different combined working conditions. The macroscopic failure modes exhibited by the specimens undergo a transition from expansion and shedding failure under low cement content conditions to swelling and tensile splitting failure under high cement content conditions. In particular, swelling failure results from the mutual compression, friction, slip and rotation of the internal aggregates in the specimen, while detachment can be attributed to the lack of bonding constraints between the aggregates after the occurrence of cement bridge cracking due to the low cement content. With a higher cement content, the overall integrity of the test specimen is better. The macroscopic failure of the specimen is primarily governed by the hardened cement, resulting in a tensile failure characteristic. As the compaction force increases, the phenomenon of aggregate detachment becomes less apparent from the specimen surface. This phenomenon could be attributed to the closer contact between aggregates achieved by higher compaction forces. Even if the cement bridge is damaged, the particles can still

interlock each other without detaching. Under the condition of high cement content, the compactness of the specimens increases with increasing specimen compaction force. The brittle fracture characteristics of the specimens become more prominent.

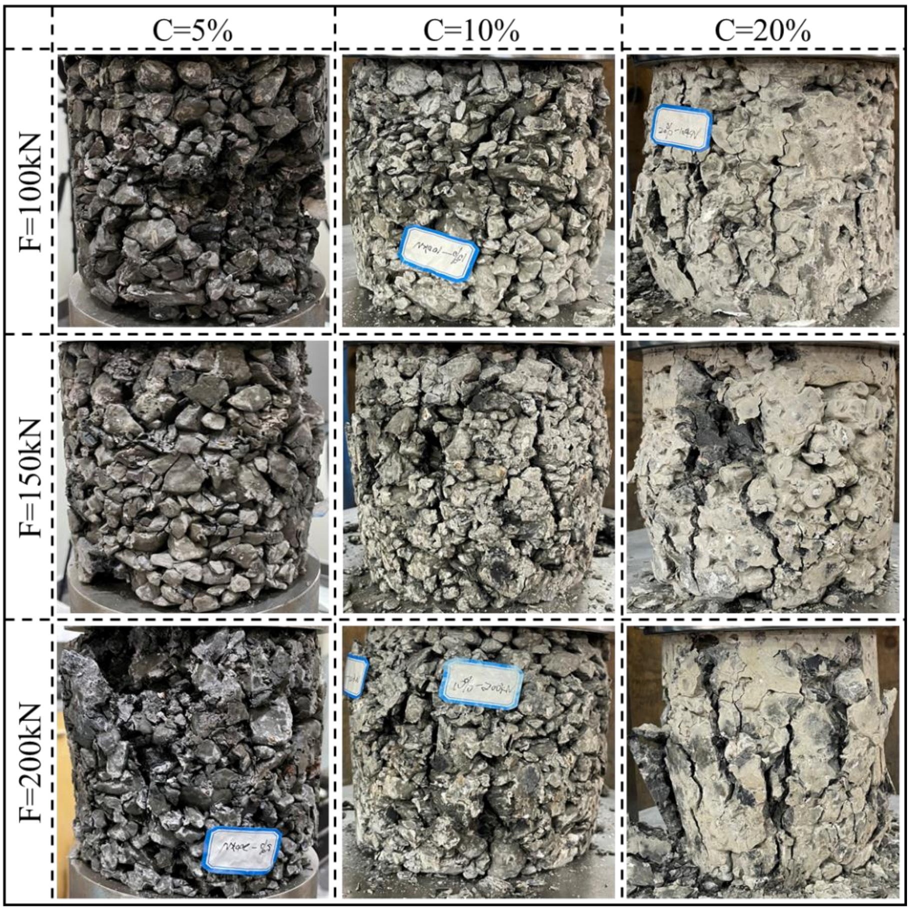

Supplementary Fig. 5 Typical failure modes of permeable cement-stabilized base material (PCBM) under different conditions

2.2 Measurement results of the porosity and permeability coefficient

Supplementary Fig. 6 displays the results of the porosity measurements of the specimens under different combination conditions. As the compaction force and cement content increase, the porosity of the specimens gradually decreases, and an increase in the cement content has a greater effect on the reduction in porosity. A lower compaction force and lower cement content are necessary preconditions to ensure that the specimen has higher porosity, but can be unfavorable for strength formation. On the other hand, a higher cement content and greater compaction force can meet the strength requirements well, but the porosity may not meet the design requirements. Therefore, from

an engineering perspective, obtaining a reasonable porosity in the specimen, a balanced cement content and compaction force are fundamental prerequisites.

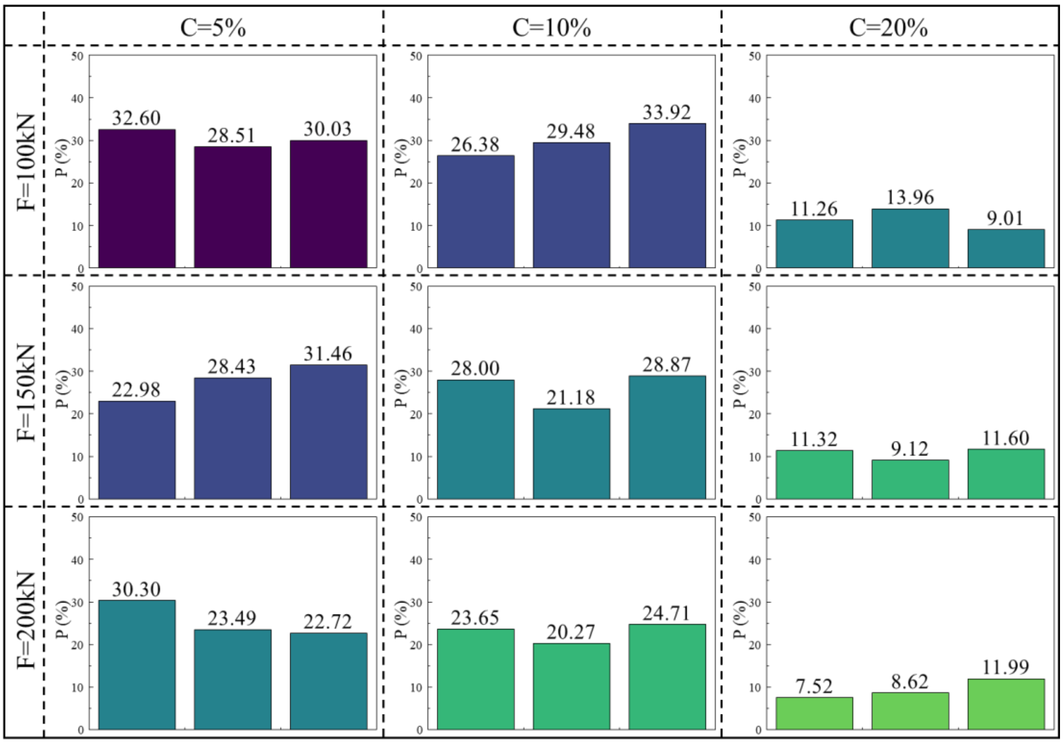

Supplementary Fig. 6 Porosity test results of the permeable cement-stabilized base material (PCBM) under different conditions

Supplementary Fig. 7 presents the test results for the permeability coefficient of the specimens under the different combination conditions. As the compaction force and cement content increase, the permeability coefficient of the specimens decreases rapidly, and when the cement content is 20%, the permeability coefficient of the specimens no longer meets the permeability requirements of the PCBM (permeability coefficient not less than 0.5 mm/s). In practical engineering, the reasonable control of the cement content is a prerequisite to ensure that the mechanical strength of PCBMs meets the standard requirements. Moreover, it is also necessary to reasonably control the compaction force to ensure the permeability characteristics of the CPBM.

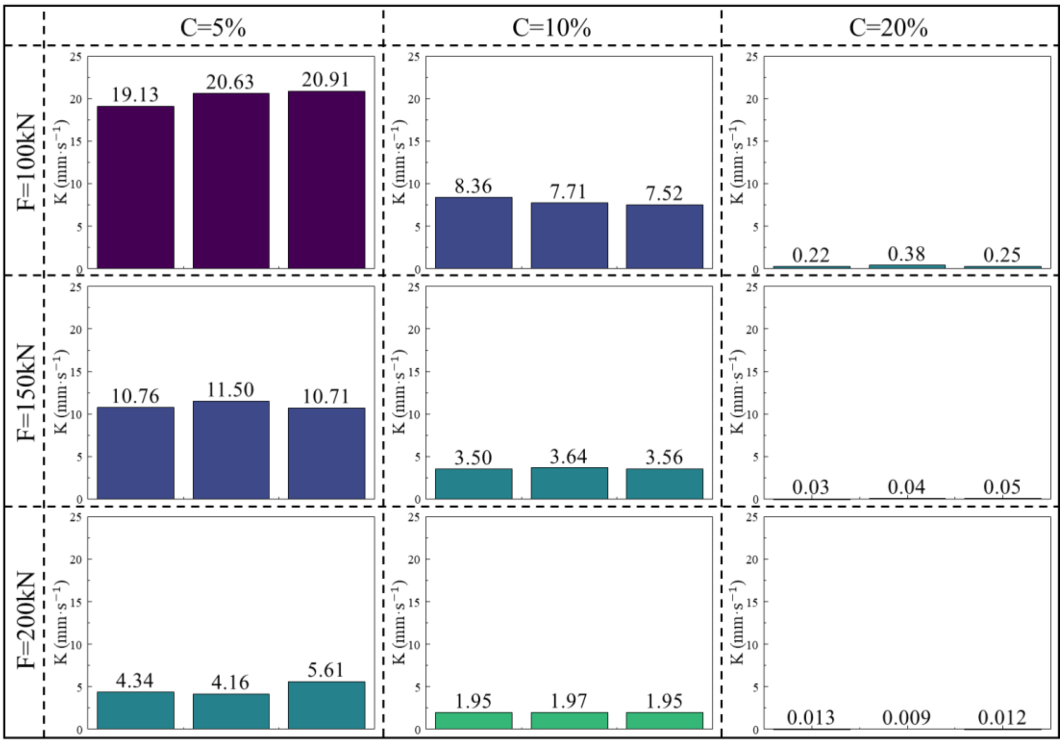

Supplementary Fig. 7 Coefficient of permeability test results of the permeable cement-stabilized base material (PCBM) under different conditions

In summary, appropriate strength is an important prerequisite for ensuring the long-term operation and durability of PCBMs in practical engineering design, while meeting the standard permeability coefficient is an important indicator for ensuring its normal drainage. However, there is currently little research on optimizing the design of PCBMs, and most methods still follow the design methods of dense cement-stabilized base materials. Conventional design methods for dense cement-stabilized base materials often focus more on the strength properties of the materials, and insufficient research has been conducted on simultaneously determining the mechanical strength and permeability coefficient.
