# Supplementary material for: Kriging-based surrogate data-enriching artificial neural network prediction of strength and permeability of permeable cement-stabilized base
Source: Nat Commun. 2024 Jun 7;15:4891. doi: 10.1038/s41467-024-48766-4 (PMC11161528; doi:10.1038/s41467-024-48766-4)
Supplement: Supplementary file 3 — Reporting Summary [file 41467_2024_48766_MOESM3_ESM.pdf]

nature portfolio

Corresponding author(s): Yuanjie Xiao

Last updated by author(s): Apr 10, 2024

Reporting Summary

Nature Portfolio wishes to improve the reproducibility of the work that we publish. This form provides structure for consistency and transparency in reporting. For further information on Nature Portfolio policies, see our Editorial Policies and the Editorial Policy Checklist.

Statistics

For all statistical analyses, confirm that the following items are present in the figure legend, table legend, main text, or Methods section.

n/a	Confirmed	
☐	☒	The exact sample size (*n*) for each experimental group/condition, given as a discrete number and unit of measurement
☒	☐	A statement on whether measurements were taken from distinct samples or whether the same sample was measured repeatedly
☒	☐	The statistical test(s) used AND whether they are one- or two-sided<br>*Only common tests should be described solely by name; describe more complex techniques in the Methods section.*
☒	☐	A description of all covariates tested
☒	☐	A description of any assumptions or corrections, such as tests of normality and adjustment for multiple comparisons
☒	☐	A full description of the statistical parameters including central tendency (e.g. means) or other basic estimates (e.g. regression coefficient) AND variation (e.g. standard deviation) or associated estimates of uncertainty (e.g. confidence intervals)
☒	☐	For null hypothesis testing, the test statistic (e.g. *F*, *t*, *r*) with confidence intervals, effect sizes, degrees of freedom and *P* value noted<br>*Give P values as exact values whenever suitable.*
☒	☐	For Bayesian analysis, information on the choice of priors and Markov chain Monte Carlo settings
☒	☐	For hierarchical and complex designs, identification of the appropriate level for tests and full reporting of outcomes
☒	☐	Estimates of effect sizes (e.g. Cohen's *d*, Pearson's *r*), indicating how they were calculated

Our web collection on statistics for biologists contains articles on many of the points above.

Software and code

Policy information about availability of computer code

Data collection	The raw data were obtained through standard laboratory tests specified for permeable cement-stabilized base materials, i.e., those tests were conducted in accordance with the requirements stated in Section 5 of the Chinese specification 'Technical Specification for Construction of Cement Stabilized Base' (DB37/T3577-2019).
Data analysis	The computer codes for Markov chain Monte Carlo simulation, kriging-based modeling, and ANN modeling were programmed and implemented using the Python program (version 3.9). The following packages were used: PyKrige (version 1.5.1), scikit-learn (version 1.1.3), numpy (version 1.23.5), pandas (version 1.5.1), and matplotlib (version 3.6.2), all of which are open source and available to the general public for free uses.

For manuscripts utilizing custom algorithms or software that are central to the research but not yet described in published literature, software must be made available to editors and reviewers. We strongly encourage code deposition in a community repository (e.g. GitHub). See the Nature Portfolio guidelines for submitting code & software for further information.

Data

Policy information about availability of data

All manuscripts must include a data availability statement. This statement should provide the following information, where applicable:

- Accession codes, unique identifiers, or web links for publicly available datasets
- A description of any restrictions on data availability
- For clinical datasets or third party data, please ensure that the statement adheres to our policy

The authors will supply the relevant data in response to reasonable requests.

Research involving human participants, their data, or biological material

Policy information about studies with human participants or human data. See also policy information about sex, gender (identity/presentation), and sexual orientation and race, ethnicity and racism.

Reporting on sex and gender	Not applicable
Reporting on race, ethnicity, or other socially relevant groupings	Not applicable
Population characteristics	Not applicable
Recruitment	Not applicable
Ethics oversight	Not applicable

Note that full information on the approval of the study protocol must also be provided in the manuscript.

Field-specific reporting

Please select the one below that is the best fit for your research. If you are not sure, read the appropriate sections before making your selection.

☐ Life sciences ☐ Behavioural & social sciences ☒ Ecological, evolutionary & environmental sciences

For a reference copy of the document with all sections, see nature.com/documents/nr-reporting-summary-flat.pdf

Ecological, evolutionary & environmental sciences study design

All studies must disclose on these points even when the disclosure is negative.

Study description	This study introduces a robust KS-ANN model that aims to enhance the prediction accuracy of machine learning on small data sets. The model's effectiveness was validated using laboratory test data of permeable cement-stabilized base material. The results demonstrate a 21% improvement in prediction accuracy as compared to the conventional ANN model, potentially leading to substantial cost savings in laboratory testing. Furthermore, the prediction results guided the optimization of design parameters for permeable cement-stabilized base material in order to enhance related engineering applications.
Research sample	In this study, the cement dosage (or proportion by weight) was chosen to vary at 5%, 10%, or 20%, while the static compaction force applied was set to vary at 100 kN, 150 kN, or 200 kN. A total of 9 different testing combinations were designed based on the orthogonal array theory, whereas a total of 27 specimens were prepared accordingly with 3 replicates for each combination of testing conditions. The unconfined compressive strength, porosity, and coefficient of permeability were measured for each specimen, thus resulting in a total of 81 original data points.
Sampling strategy	A hybrid adaptive sampling strategy was used in performing kriging-based surrogate modeling.
Data collection	The raw data were obtained through standard laboratory tests specified for permeable cement-stabilized base materials. Those tests were conducted in accordance with the requirements stated in Section 5 of the Chinese specification 'Technical Specification for Construction of Cement Stabilized Base' (DB37/T3577-2019). First of all, cement, water, and aggregates were mixed and compacted in the mold according to the designed proportions. The compacted specimens were then left to stand for 24 hours and transferred to the standard curing room for a duration of 28 days. After the completion of curing, the porosity, coefficient of permeability, and unconfined compressive strength of the specimens were tested subsequently. The following steps were followed to measure the porosity: (1) measuring the dry weight of the specimen three times and recording the data; (2) suspending the specimen with a fine line and completely immersing it in water, then measuring the buoyant gravity of the specimen every 5 minutes for a total of three times and recording the data; and (3) calculating the porosity of the specimen using the related formula. The steps for measuring the coefficient of permeability were as follows: (1) placing the specimen in the permeameter and ensuring it was securely positioned against the wall of the instrument; (2) turning on the water faucet to completely submerge the specimen and reach the desired head height. Once the water flowing under the instrument stabilized (approximately 10 minutes), measuring the flow rate of the outflow water started; (3) taking three measurements of the outflow water flow rate on one side at 3-minute intervals and recording the data; and (4) calculating the coefficient of permeability of the specimen using the recorded flow rate and the dimensions of the

instrument. The steps for measuring the unconfined compressive strength are as follows: (1) positioning the specimen, which has undergone necessary maintenance, into the compactor with a maximum force range of 2000 kN. Adjusting the position so that the loading piston and platen are in contact with the specimen; (2) applying compressive pressure to the specimen at a loading speed of 50 mm/min until it was completely damaged; (3) recording the peak strength upon specimen failure as the unconfined compressive strength.

Timing and spatial scale	Not applicable
Data exclusions	Not applicable
Reproducibility	The laboratory test results presented in this study can be replicated by following the guidelines provided in the Chinese specification 'Technical Specification for Construction of Cement Stabilized Base' (DB37/T3577-2019). To validate the effectiveness of the proposed KS-ANN model, a conventional ANN model was established and used for comparison. The results indicate an overall improvement of 21% in the prediction accuracy of the KS-ANN model when applied to small datasets. In order to facilitate reproduction of the KS-ANN model, a detailed description of the methodology is provided in this article for reference.
Randomization	In this paper, the test results of multiple samples prepared using a set of design parameters (cement content and static compaction force) are random. Therefore, this paper characterizes the random distribution of test results corresponding to a set of design parameters. Therefore, when a sufficient number of random numbers are specified in the KS-ANN model, the input set of design parameters can predict the statistical distribution of test results.
Blinding	Blinding was not implemented in this study as it solely consisted of quantitative trials and predictive analysis of data.

Did the study involve field work? ☐ Yes ☒ No

Reporting for specific materials, systems and methods

We require information from authors about some types of materials, experimental systems and methods used in many studies. Here, indicate whether each material, system or method listed is relevant to your study. If you are not sure if a list item applies to your research, read the appropriate section before selecting a response.

Materials & experimental systems

n/a	Involved in the study
☒	☐ Antibodies
☒	☐ Eukaryotic cell lines
☒	☐ Palaeontology and archaeology
☒	☐ Animals and other organisms
☒	☐ Clinical data
☒	☐ Dual use research of concern
☒	☐ Plants

Methods

n/a	Involved in the study
☒	☐ ChIP-seq
☒	☐ Flow cytometry
☒	☐ MRI-based neuroimaging

Plants

Seed stocks	Not applicable
Novel plant genotypes	Not applicable
Authentication	Not applicable
